# Examining changes in personality disorder and symptomology in an adolescent sample receiving intensive mentalization based treatment: a pilot study

**DOI:** 10.1186/s13034-017-0197-9

**Published:** 2017-11-28

**Authors:** Kirsten Hauber, Albert Eduard Boon, Robert Vermeiren

**Affiliations:** 1 De Jutters B.V, Centre for Youth Mental Healthcare Haaglanden, The Hague, The Netherlands; 2 Lucertis, Child and Adolescent Psychiatry Rotterdam, Rotterdam, The Netherlands; 3 Department of Child and Adolescent Psychiatry, Curium-Leiden University Medical Centre, Leiden, The Netherlands; 4 Department of Child and Adolescent Psychiatry, VU University Medical Centre, Amsterdam, The Netherlands

## Abstract

**Objective:**

To examine changes in personality disorders and symptomology and the relation between personality disorder variables and treatment outcomes in an adolescent sample during partial residential mentalization based treatment.

**Methods:**

In a sample of 62 (out of 115) adolescents treated for personality disorders, assessment was done pre- and post-treatment using the Structured Clinical Interview for DSM personality disorders and the Symptom Check List 90.

**Results:**

Significant reductions in personality disorder traits (*t* = 8.36, *p* = .000) and symptoms (*t* = 5.95, *p* = .000) were found. During pre-treatment, 91.8% (*n* = 56) of the patients had one or more personality disorders, compared to 35.4% (*n* = 22) at post-treatment. Symptom reduction was not related to pre-treatment personality disorder variables.

**Conclusion:**

During intensive psychotherapy, personality disorders and symptoms may diminish. Future studies should evaluate whether the outcomes obtained are the result of the treatment given or other factors.

## Background

Relatively little research has been conducted on personality disorders in adolescents; specifically, research regarding effective treatments is limited [1–5]. This is an omission, as the psychosocial and the economic burdens of adolescents with (traits of) personality disorders are high [3, 6]. Interestingly, the direct mental health and medical costs for adolescents in the year prior to treatment for personality disorders were demonstrated to be substantially higher than for adults [6, 7]. Timely detection and treatment of (traits of) personality disorders during adolescence are for that reason important. Therefore, the aim of this cohort pilot study is to examine the changes in a group of adolescents with clinically diagnosed personality disorders who received an intensive mentalization based treatment (MBT) with partial hospitalisation [8–10]. Mentalizing refers to the ability to understand and differentiate between the mental states of oneself and others and to acknowledge the relation between underlying mental states and behaviour [8, 11].

Doubts regarding the permanence of personality disorders in adolescents are considered to be the main problem underlying the lag in research on this topic [2, 3, 12, 13]. Despite guidelines [14] advising professionals to diagnose personality disorders (with the exception of antisocial personality disorder during adolescence), most psychologists and psychiatrists are hesitant to diagnose personality disorders in minors. As a result, minors are not offered specific treatments. This is partly understandable as, during adolescence, normal emotional maturation is characterised by an interplay between progression and regression [15], which complicates the diagnostic process of personality disorders. In addition, diagnosing personality disorders might stigmatise adolescents. However, the reluctance of professionals to diagnose (traits of) personality disorders in adolescents is likely to delay research and thus the development of effective treatments for this group of patients.

According to current research, the primary information used to treat personality disorders in adolescents is based on randomised controlled trials of treatments developed for adults, mostly treatments for borderline personality disorder (BPD). The few studies that have been conducted on adolescents with (traits of) BPD have yielded mixed results. Two studies showed no advantages over treatment as usual [16, 17]; one study showed only a short term effect [18]; while another found a better outcome compared to treatment as usual [19]. All treatments were associated with improvements over time, which may partially reflect the natural course of BPD in adolescents. Whether existing adult treatment programmes are useful for adolescents with personality disorders other than BPD is mostly unknown, as research is scarce. One study investigated the treatment outcome of a 12 month inpatient psychotherapy intervention for adolescents with personality disorders. Only 51 patients of a total sample of 109 completed the research protocol, of whom 29% recovered fully in terms of the level of symptom severity, 12% improved, while 49% showed no significant change and 10% showed deterioration [20]. Furthermore, none of the specific personality disorders or clusters of personality disorders (A, B, C and NOS) predicted treatment outcome. In conclusion, the results of the few studied treatments for adolescents with (traits of) personality disorders have shown mixed results; however, the most severe sample studied, the inpatient group, showed moderate results.

Difficulties in establishing randomised clinical trials (RCTs) in clinical practice—especially in a high risk adolescent sample with comorbidity—is another reason that potentially explains the scarcity of research in adolescents with personality disorders. Although RCTs are essential for studying the comparative effectiveness of treatments and have a high internal validity, trials dictate strict protocol adherence and often have a low external validity [21]. Furthermore, randomising carries ethical and practical ramifications in a high risk adolescent group in need of an inpatient programme due to family dynamics, suicidal actions, self-injury and prolonged school absenteeism. Randomisation on the individual level within an inpatient treatment programme is even more intricate, as it implies training half of the treatment staff to follow a study protocol and compare the effect of their interventions with the effect of the interventions of the non-trained half. Moreover, as populations and circumstances differ significantly, the results of RCTs may have limited relevance to clinical practice. Therefore, nonrandomised evaluations of inpatient programmes focusing on external validity, in order to obtain generalisable knowledge of the patient group and treatment evaluation, are needed. The transparent reporting of evaluations with nonrandomised designs (TREND) group [22] has developed a 22 items checklist to improve the reporting standards of nonrandomised evaluations of behavioural and public health interventions.

In this study, we provide treatment evaluation data following the TREND guidelines [22] from a prospective pilot study of 115 adolescents with clinically diagnosed personality disorders, of whom 62 (54%) completed the treatment protocol and filled out questionnaires during pre- and post-treatment. This group received intensive MBT with partial hospitalisation [8–10]. The external validity is tested. Furthermore, the predictive power of personality disorder variables on treatment outcomes concerning symptomology is explored.

## Methods

### Setting

The present study was conducted from January 2008 until December 2014 at a residential psychotherapeutic institution for adolescents in the urban area of The Hague in the Netherlands. This facility offers a 5 days a week intensive MBT with partial hospitalisation for adolescents between the ages of 16 and 23 years with personality disorders. This structured and integrative psychodynamic group psychotherapy programme is manualised, adapted to adolescents [8–10] and facilitated by a multidisciplinary team trained in MBT. The major difference with the MBT programme for adolescents in England [19] is the psychodynamic group psychotherapy approach. The mentalizing focus of the different therapies in the programme is on the adolescent’s subjective experience of himself or herself and others and on the relationships with the group members and therapists. The programme offers weekly verbal and non-verbal group psychotherapies, such as group psychotherapy, art therapy and psychodrama therapy, in combination with individual and family psychotherapy. The average duration of treatment is 1 year with a maximum of 18 months. Commonly, the treatment starts with hospitalisation and continues as day treatment later on during the programme. Medication is prescribed if necessary by a psychiatrist working in the therapy programme, according to protocol. Referrals come non-systematically from other mental health professionals from within and outside our mental health care institution.

### Subjects

In total, 115 adolescents with clinically diagnosed personality disorders were studied with a mean age at the start of treatment of 18.2 (*SD* = 1.6, range = 15–22; females 80.9%). Most of the participants had other comorbid axis-I disorders (mood disorder 58%; anxiety disorder, including PTSD 31%; eating disorder 13%; ADHD 8%; substance dependence 7%; dissociative disorder 3%; and obsessive compulsive disorder 2%). The average duration of treatment was 277.8 days (*SD* = 166.1, range = 3–549), with an average of 186.1 days (*SD* = 146.1) of hospitalisation. Intelligence was estimated based on the level of education and was average to above average. All patients followed the treatment on a voluntary basis and were fluent in the Dutch language.

Of the 115 adolescents who were included in this study, 13 were considered treatment dropouts because they withdrew or were sent away before their treatment duration exceed the diagnostic phase of 2 months (61 days) [23, 24]. These 13 dropouts did not differ significantly from the rest in age, gender or severity of symptoms or personality disorders. The remaining sample consisted of 102 respondents, with 83 females (81.4%) and 19 males (18.6%). While all were assessed by the SCID-II interview initially, only 62 (60.8%) post-treatment SCID-II interviews were administered. One adolescent did not complete the SCID-II interview at pre-treatment but did at post-treatment. The average duration of treatment of adolescents who only participated in a pre-treatment SCID-II interview was shorter (202.1 days; *SD* = 115.2, 61–526), with an average of 146.4 (*SD* = 124.9, 0–20) days of hospitalisation, compared to those who also participated in a post-treatment SCID-II interview (378.6 days; *SD* = 126.0, 120–549), with an average of 246.0 (*SD* = 139.4, 0–547) days of hospitalisation (*p* = 0.000; *t* = 7.406). Of the respondents who only participated in a pre-treatment SCID-II interview, 43% completed the treatment according to protocol, as compared to 92% of the adolescents who also participated in a post-treatment SCID-II interview. The number and type of personality disorders did not differ between these groups. Missing post-treatment research data was caused by respondents who failed to complete the set of web-based questionnaires during post-treatment or repeatedly failed to show up at the final SCID-II interview appointment.

### Measures

The participating adolescents completed a set of web-based questionnaires at the beginning and end of treatment, including the Dutch Questionnaire for Personality Characteristics (Vragenlijst voor Kenmerken van de Persoonlijkheid) (VKP) [25] and the Symptom Check List 90 (SCL-90) [26, 27]. Subjects were interviewed using the Structured Clinical Interview for DSM personality disorders (SCID-II) [28].

#### VKP

The VKP is a questionnaire consisting of 197 questions with the answer categories ‘true’ or ‘false’; its purpose is to screen for personality disorders according to the DSM-IV. The VKP is known for its high sensitivity and low specificity [25] and is recommended [29, 30] as a pre-assessment instrument before administering the Dutch version of the SCID-II. Presumed and certain indications of a personality disorder on the VKP indicate which SCID-II personality disorder sections should be applied. The test–retest reliability (Cohen’s Kappa) of the VKP on categorical diagnoses was moderate (*k* = .40) [25].

#### SCL-90

An authorised Dutch version of the SCL-90 [26] is a questionnaire consisting of 90 questions with a 5-point rating scale (ranging from 1 ‘not at all’ to 5 ‘extreme’). This questionnaire assesses general psychological distress and specific primary psychological symptoms of distress. Outcome scores are divided into nine symptom subscales: anxiety; agoraphobia; depression; somatisation; insufficient thinking and handling; distrust and interpersonal sensitivity; hostility; sleeping disorders; and a rest subscale. The total score (range 90–450) is calculated by adding the scores of the subscales. The test–retest reliability was reasonable to good (*k* = .62 to .91) [26].

#### SCID-II

The SCID-II [28] is a semi-structured interview consisting of 134 questions. The purpose of this interview is to establish the ten DSM-IV personality disorders, and depressive and passive-aggressive personality disorders. In line with the DSM-IV criteria, the depressive and passive-aggressive personality disorders are covered by the ‘personality disorder not otherwise specified’ (NOS). The language and diagnostic coverage make the SCID-II most appropriate for adults (age 18 or over), while with slight modification it can be used for younger adolescents [28]. Only the sections that were indicated by the outcome of the VKP were applied in the clinical interview. The SCID-II was administered by trained psychologists. The inter-rater reliability (Cohen’s Kappa) of the SCID-II for categorical diagnoses was reasonable to good (*k* = .61–1.00) [31], and the test–retest reliability was also reasonable to good (*k* = .63) [32].

### Procedures

From 2008, 115 newly admitted patients were asked to participate in the study. The data of patients ending treatment before the end of 2014 were used. Following a verbal description of the treatment protocol to the subjects, written informed consent was obtained according to legislation, the institution’s policy and the Dutch law [33]. All patients (*N* = 115) agreed to participate and, in accordance with the institutional policy, they participated without receiving incentives or rewards. All procedures in this study were in accordance with the 1964 Declaration of Helsinki and its later amendments or comparable ethical standards. According to the treatment protocol, the patients completed a set of web-based questionnaires, including the VKP and the SCL-90 during the first and last weeks of treatment. The participants filled out the questionnaires by themselves and were not aware of the study’s objective.

### Statistical analysis

All analyses were performed using the Statistical Package for the Social Sciences, version 20.0 [34]. A Wilcoxon Signed-Rank Test was performed between the number of pre-treatment SCID-II personality disorders and the number of post-treatment SCID-II personality disorders. To compare the total score on the SCL-90 across the number of SCID-II personality disorders at pre- and post-treatment an ANOVA was used. A Pearson correlation test was performed to compare the length of treatment with changes in the SCL-90 and paired *t* test were performed to compare the SCL-90 and number of SCID- II personality disorders between two groups based on length of treatment. A linear regression analysis was used to explore the relationship between the predictor variables (VKP, SCID-II scales) at t − 1 and the SCL-90 outcome at post-treatment.

## Results

### Pre- and post-treatment personality disorders SCID-II

In Table 1, the number of patients who met the criteria for a personality disorder according to the VKP and the SCID-II at pre- and post-treatment are shown.

**Table 1:** Number of patients with personality disorders according to the VKP and the SCID-II at *t* − 1 and *t* − 2 (N = 62)

	*t* − 1				*t* − 2			
	VKP*		SCID-II		VKP*		SCID-II	
	N	%	N	%	N	%	N	%
No PD	3	4.8	6	9.7	15	24.2	40	64.5
Paranoid PD	31	50.0	13	20.9	11	17.7	5	8.1
Schizoid PD	11	17.7	2	3.2	3	4.8	0	0.0
Schizotypal PD	12	19.4	0	0.0	1	1.6	0	0.0
Antisocial PD	6	9.7	1	1.6	1	1.6	0	0.0
Borderline PD	18	29.0	23	37.1	5	8.1	7	11.3
Histrionic PD	4	6.4	0	0.0	2	3.2	0	0.0
Narcissistic PD	1	1.6	0	0.0	0	0.0	0	0.0
Avoidant PD	41	66.1	34	54.8	19	30.6	11	17.7
Dependant PD	19	30.7	3	4.8	6	9.7	1	1.6
Obsessive compulsive PD	15	24.2	8	12.9	5	8.1	3	4.8
Depressive PD	32	51.6	29	46.8	8	12.9	9	14.5
Passive aggressive PD	5	8.1	2	3.2	2	3.2	0	0.0
PD NOS			2	3.2			1	1.6

*PD* personality disorder

* Certain indications of a personality disorder according to the VKP. The presumed indications of a personality disorder according to the VKP were left out of this table

When comparing the number of pre-treatment versus post-treatment SCID-II personality disorders, a significant decrease was found (*t* − *1*: *M* = 1.42, *SD* = 1.21, range 0–4; *t* − *2*: *M* = 0.48, *SD* = 0.78, range 0–4; *z* = 5.76, *p* = .000). The effect size for this analysis (*d* = 0.92, 95% CI [0.77–1.26]) was found to exceed Cohen’s (1988) convention for a large effect (*d* = .80). At pre-treatment, 91.8% (*n* = 56) of the patients had one or more personality disorders, compared to 35.4% at post-treatment (*n* = 22). The majority, 74.1% (*n* = 46) of patients, showed a decrease in the number of SCID-II personality disorders at the end of treatment; 19.4% (*n* = 12) retained the same number; and 6.5% (*n* = 4) had more personality disorders at the end of the treatment. Although clinical judgment indicated a personality disorder, at the start of treatment, six (9.6%) patients were free of any personality disorder on the SCID-II. One adolescent out of the six deteriorated to having one SCID-II personality disorder at the end.

### Pre- and post-treatment personality disorders and SCL-90

**Fig. 1:**
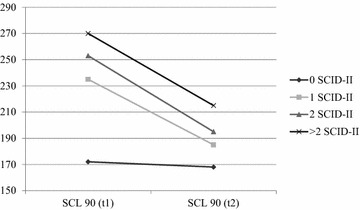
Comparison of the pre- and post-treatment total SCL-90 score by number of SCID-II diagnosis initially

Of the 62 adolescents who participated in pre- and post-treatment SCID-II interviews, 56 (90.3%) completed the SCL-90 at both points in time. A significant symptom reduction was observed (*t* = 5.95, *p* = .000). The mean t − 1 total score of 241.0 (*SD* = 51.8) on the SCL-90 declined to 189.8 (*SD* = 64.8) at t − 2 (*d* = .87, 95% CI [33.9–68.4]). A significant correlation was found at pre- and post-treatment between the number of SCID-II personality disorders and the total score on the SCL-90 (*t* − *1: N* = 61, *F* = 4.71, *p* = .005; *t* − *2: N* = 57, *F* = 10.64, *p* = .000) (Fig. 1).

The group with one or more SCID-II personality disorders (*n* = 51) differed significantly on the total SCL-90 score between pre- (247.73, *SD* = 47.38) and post-treatment (191.92, *SD* = 63.77; *t* = 6.29, *p* = .000, *d* = .87, 95% CI [35.9–68.7]). Moreover, the separate groups of SCID-II personality disorders reported significantly fewer symptoms at post-treatment in comparison to their initial levels (Table 2). The group without SCID-II personality disorders at the start of treatment reported fewer symptoms both pre- and post-treatment in comparison to the SCID-II groups, and it showed no symptom decrease (*n* = 5, *t* − *1*: 172.20, *SD* = 48.90; *t* − *2*: 168.20, *SD* = 78.84, *t* = 0.15, *p* = .891, *d* = .06, 95% CI [− 72.2 to 80.2]).

**Table 2:** Comparison of the number of personality disorders at the start with the total SCL-90 score pre- and post-treatment

Number of personality disorders at *t* − *1*		Total SCL-90 score					
	*n*	*t* − *1*		*t* − *2*		*t*	*p*
		Mean	*SD*	Mean	*SD*		
0	5	172.20	48.90	168.20	78.84	0.15	.891
1	29	240.31	51.39	187.07	60.05	4.27	.000
2	16	255.25	40.39	198.25	70.18	3.61	.003
> 2	6	263.50	44.38	198.50	73.40	3.04	.029

### Length of treatment and changes in the SCL-90 and the SCID-II

No significant correlation was found between the length of treatment and symptom reduction on the total SCL-90 (*r* = 0.168; n = 64; *p* = .184). The total group was divided in three groups based on length of treatment, resulting in a less than 234 days group (N = 8), a 235–364 days group (N = 22) and a more than 365 days group (N = 32). The less than 234 days group (N = 8) was to small for analyses and had to be excluded. The two remaining groups based on length of treatment, the 235–364 days group and the more than 365 days group, were compared by using the total SCL-90 scores and the number of SCID-II personality disorders at the beginning and the end of treatment. The 235–364 days group (symptoms: *n* = 23, *t* − *1*: 233.00, *SD* = 47.76; *t* − *2*: 190.87, *SD* = 61.44, *t* = 3.68, *p* = .001, *d* = .77; personality disorders: *n* = 22, *t* − *1*: 1.73, *SD* = 1.03; *t* − *2*: .59, *SD* = .73, *t* = 4.74, *p* = .000, *d* = 1.28) and the more than 365 days (symptoms: *n* = 31, *t* − *1*: 247.45, *SD* = 55.16; *t* − *2*: 183.84, *SD* = 64.21, *t* = 5.15, *p* = .000, *d* = 1.06; personality disorders: *n* = 32, *t* − *1*: 1.97, *SD* = 1.23; *t* − *2*: .63, *SD* = 1.16, *t* = 6.29, *p* = .000, *d* = 1.12) showed approximately equal symptom and number of personality disorders reduction. No significant differences were found between the two length of treatment groups on the different SCID-II personality disorders.

### Predictive value of personality disorder variables on treatment outcome

The scales of the pre-treatment VKP and pre-treatment SCID-II were entered in a logistic regression with age, gender and duration of treatment as control variables and SCL-90 outcome as a dependent variable. None of the independent variables contributed significantly to the outcome.

## Discussion

Our pilot study indicates that, during intensive psychotherapeutic treatment including partial hospitalisation, the number of personality disorders and symptoms may decrease substantially. At the end of the treatment, approximately three quarters of the participants showed a lower number of personality disorders, while two-thirds did not meet the SCID-II criteria for a personality disorder after treatment any longer. However, a large part of the sample was not assessed at the end of the treatment. Since this cohort study was not randomised, it is not possible to draw conclusions about the direct effect of the treatment itself. Furthermore, symptom reduction could not be predicted by pre-treatment personality disorder variables. Nevertheless, this pilot study suggests that personality disorders in adolescents can diminish during intensive psychotherapy.

It is of substantial clinical interest to examine whether the positive outcome obtained in the part of the sample that completed measurements at t − 1 and t − 2 was the result of the provided treatment or other factors. Age-related development or the social support of family and friends [35] may partly have been responsible for the decrease in symptoms and personality pathology. Nevertheless, if the treatment affected the outcome, focus should be placed on examining which element of the treatment caused these improvements. A hypothesis is that working in a group with a group psychodynamic approach is especially relevant for adolescents [36]. In combination with MBT [8–10] and the focus on the relationships with group members and therapists, this may have stimulated a positive outcome. Future research directions should focus on the role of treatment groups for adolescents with personality disorders in treatment outcomes.

Moreover, the duration of the partial hospitalisation may be a factor of particular relevance. The treatment lasted relatively long, and effects of time cannot be ruled out without a control group. The effectiveness of approximately 5 months inpatient psychotherapeutic treatment was described as optimal for adults with cluster B personality disorders [37], cluster C personality disorders [38] and with personality disorders not otherwise specified [39], in comparison to longer inpatient psychotherapeutic treatment. Currently, the maximum duration of partial hospitalisation is set at 6 months. Future research should examine whether there is a general optimal duration of hospitalisation for an intensive group psychotherapy programme for adolescents with personality disorders or the variables a personal optimal length depends on.

Considering our results, the question is whether adolescents with personality disorders are more capable of change than adults with similar problems, as our study found larger changes than those observed in most adult studies. Developmental change may have played a role, as it is known that adolescents become more capable of regulating emotions and behaviour over time. Adolescence may be a developmental phase in which opportunities for change in personality pathology are greater, under the right conditions, than in adulthood. Furthermore, clinical impression suggests that joint problem definition between parents and adolescents, willingness to change and parental support, together with a relatively stable and safe home environment, are crucial to the treatment’s success. These factors may be of less crucial importance in adults. If parents are not able to reflect on family dynamics and are critical towards treatment offers, the treatment has fewer chances of success. Unfortunately, in this study no data were collected regarding the role of parents. Future research should examine the effect of the role of parents on the treatment outcome in adolescents with personality disorders.

It is necessary to discuss the strengths and limitations of this study. One strength was the inclusion of a high risk adolescent sample with comorbidity that is rarely examined. The first limitation is that only part of the patients that were included in this study could be followed from the start until the end of treatment. Information about the patients we did not follow is scarce. Initially, however, these patients did not differ in number and type of personality disorders. The shorter duration of treatment suggests that this group either profited less from treatment than those who completed it or improved enough so as not wish to continue treatment. In this study, possible causal mechanisms for the premature termination of therapy amongst adolescents with personality disorders remained unclear. The second shortcoming of this study was that the Axis I disorders were left out due to the practical consideration of not overloading patients with assessment instruments. Finally, the third limitation is that, due to the research design, the extent to which treatment played a role in the positive outcome and which parts of the programme may have contributed remains unknown.

Research on the outcome of treatment for adolescents with personality disorders other than borderline personality disorder or a combination of personality disorders is scarce [5]. Examining the specific mechanisms of change in the different treatments for adolescents with personality disorders is thus important. The treatment examined in this pilot study is promising, although essential questions remain unanswered. Replication is necessary in order to determine whether the results were based on coincidence or not.
